# Personal

**Published:** 1915-08

**Authors:** 


### PERSONAL.

**Announcement of removal, travel, and other matters of interest are requested. Please report errors in the listing of any physician in the State and other directories, that we may co-operate with the State Society in securing a correct list.**

Dr. Roger S. Morris of Clinton Springs, has been appointed to the recently established Forchheimer Chair of Internal Medicine at the University of Cincinnati.

Dr. H. Sheridan Baketel, editor of the Medical Times of N. Y., has been appointed chief of the department of Hygiene and Public Health of the Long Island College Hospital.

Dr. George W. Crile of Cleveland, gave a clinic at the Good Shepard Hospital at Syracuse, in June.

Dr. Isaac W. Brewer, recently married to Miss Charlotte Underhill of Syracuse, where he formerly resided, has moved to Geneva. He is one of the State District Sanitary Supervisors.

Dr. Albert VanderVeer of Albany, is President-elect of the A. M. A.

Dr. Henry Elsner of Syracuse, has received the degree of LL.D. from the University of Syracuse.

Dr. A. G. Dunbar, Buffalo 1912, is Superintendent of the Oswego Co. Tuberculosis Hospital. He is practicing in Richland.

Dr. Mary Innis Denton, formerly a practitioner of Buffalo, is in Buffalo for the summer at 253 Rhode Island St.

Dr. Richard G. Wright of Buffalo collided with a Niagara Falls trolley in N. Tonawanda July 18. The automobile was damaged but the occupants were not seriously hurt.

Dr. Edward L. Frost of Buffalo attended the commencement of the University of Michigan, where his son Carl, received the medical degree.

The automobile of Dr. Irving W. Potter of Buffalo was stolen from in front of Dr. Harry Mead's residence, early in July and was badly damaged in a collision with a taxicab.

Dr. George Edward Fell has recovered his health and has located at Carlton Court, Main and Carlton Sts., Buffalo.

A bicycle ran into the automobile of Dr. M. F. Nolan of N. Tonawanda, the same day, wrecking the bicycle but resulting in only slight injury to the rider.

